# S34. DEVELOPMENTAL TRAJECTORIES OF PSYCHOTIC EXPERIENCES AND THEORY OF MIND IN 11-YEAR-OLD OFFSPRING OF PARENTS WITH SCHIZOPHRENIA OR BIPOLAR DISORDER

**DOI:** 10.1093/schbul/sby018.821

**Published:** 2018-04-01

**Authors:** Maja Gregersen, Lars Clemmensen, Anne Søndergaard, Camilla Jerlang Christiani, Nicoline Hemager, Ditte Ellersgaard, Katrine Søborg Spang, Aja Greve, Ditte Lou Gantriis, Christina Bruun Knudsen, Anna Krogh Andreassen, Lotte Veddum, Henriette Brockdorff Stadsgaard, Vibeke Fuglsang Bliksted, Ole Mors, Kerstin J Plessen, Merete Nordentoft, Jens Richardt Møllegaard Jepsen, Anne A E Thorup

**Affiliations:** 1 Mental Health Services, Capital Region of Denmark, Mental Health Centre Copenhagen; 2 Mental Health Services, Capital Region of Denmark, Child and Adolescent Mental Health Centre; 3 Mental Health Services, Capital Region of Denmark, Mental Health Centre Copenhagen, The Lundbeck Foundation Initiative for Integrative Psychiatric Research; 4 Mental Health Services, Capital Region of Denmark, Mental Health Centre Copenhagen, Child and Adolescent Mental Health Centre, The Lundbeck Foundation Initiative for Integrative Psychiatric Research; 5 Mental Health Services, Capital Region of Denmark, Child and Adolescent Mental Health Centre, The Lundbeck Foundation Initiative for Integrative Psychiatric Research; 6 The Lundbeck Foundation Initiative for Integrative Psychiatric Research, Aarhus University Hospital; 7 Mental Health Services, Capital Region of Denmark, Child and Adolescent Mental Health Centre, The Lundbeck Foundation Initiative for Integrative Psychiatric Research, University of Copenhagen; 8 Mental Health Services, Capital Region of Denmark, Mental Health Centre Copenhagen, The Lundbeck Foundation Initiative for Integrative Psychiatric Research, University of Copenhagen; 9 Mental Health Services, Capital Region of Denmark, Child and Adolescent Mental Health Centre, The Lundbeck Foundation Initiative for Integrative Psychiatric Research, Center for Clinical Intervention and Neuropsychiatric Schizophrenia Research

## Abstract

**Background:**

The study is a part of the Danish High Risk and Resilience Study, Via 11 and aims to explore the developmental trajectories of psychotic experiences (PEs) and theory of mind in children born to parents with schizophrenia or bipolar disorder. In a cross sectional perspective we also aim to explore possible associations between PEs and social cognitive deficits, particularly hyper-theory-of-mind. We also wish to explore the significance of other potential risk factors for PEs such as cognitive biases, adverse life events, and insecure attachment styles. Earlier studies have shown that PEs during childhood are predictive of later psychotic disorders, especially if they persist over time. We expect the possible risk factors to have a cumulative effect.

**Methods:**

The Danish High Risk and Resilience Study, Via 11, is the first follow-up of a cohort of 522 children and their parents. The cohort consists of children where one or both parents have been diagnosed with a schizophrenia spectrum disorder (N=202), children where one or both parents have been diagnosed with bipolar affective disorder (N=120) and children where neither of the parents have been diagnosed with these disorders (N=200). The children and their parents were assessed with a comprehensive assessment battery e.g. social- and neurocognitive tests and diagnostic interviews when the children were seven years old, and they will now be re-assessed for the first time at age 11. Data for this study is currently being collected as a part of the Via 11. Psychotic experiences will be assessed on the Scale of Prodromal Symptoms based on K-SADS interviews and with the Magical Thinking Questionnaire. Social cognitive skills will be assessed with Frith-Happé Animated Triangles and Theory-of-Mind Storybook Frederik. Cognitive bias i.e. jumping to conclusions will be assessed with the Beads task. Adverse life events will be assessed with the K-SADS interviews, the Child Trauma Screening Questionnaire, and with a questionnaire about bullying based on the Olweus Bully/Victim Questionnaire. Measures of neurocognitive and attentional deficits will also be included. Child attachment style was assessed with the Story Stem Assessment Protocol and emotion recognition with the ERT from Cantab.

Hypotheses:

-Age seven: Children in the two high risk groups will be have higher rates of insecure or disorganized attachment styles compared with children in the control group. We expect insecure and disorganized attachment to be associated with poorer social cognition (theory of mind and emotion recognition) and with worse general psychopathology and PEs.

-Age 11: We expect children born to parents with schizophrenia spectrum disorders to report higher frequencies of PEs than children born to parents without these disorders. We expect children with PEs to have higher levels of general psychopathology and poorer levels of daily functioning than children without PEs. We expect children in the two high risk groups to have poorer theory of mind than children in the control group.

-Age 11: We expect PEs to be associated with poor social cognition, particularly hyper theory-of-mind, higher rates of cognitive bias, adverse life events, neurocognitive and attentional impairments, and to be predicted by insecure and disorganized attachment styles.

**Results:**

The data collection started in March 2017. Results from the 11-year-follow-up are expected in 2020.

**Discussion:**

Examining PEs over time during childhood is important since it may improve our ability to identify children who are at a particularly high risk of developing psychotic disorders and other psychopathology later in life and thus to identify a particularly vulnerable subgroup towards whom early interventions should be targeted.

